# GRPR versus PSMA: expression profiles during prostate cancer progression demonstrate the added value of GRPR-targeting theranostic approaches

**DOI:** 10.3389/fonc.2023.1199432

**Published:** 2023-08-31

**Authors:** Marjolein Verhoeven, Eline A. M. Ruigrok, Geert J. L. H. van Leenders, Lilian van den Brink, Hayri E. Balcioglu, Wytske M. van Weerden, Simone U. Dalm

**Affiliations:** ^1^ Department of Radiology & Nuclear Medicine, Erasmus MC, University Medical Center Rotterdam, Rotterdam, Netherlands; ^2^ Department of Pathology, Erasmus MC Cancer Institute, University Medical Center Rotterdam, Rotterdam, Netherlands; ^3^ Department of Medical Oncology, Erasmus MC, University Medical Center Rotterdam, Rotterdam, Netherlands; ^4^ Department of Experimental Urology, Erasmus MC, University Medical Center Rotterdam, Rotterdam, Netherlands

**Keywords:** gastrin-releasing peptide receptor (GRPR), prostate-specific membrane antigen (PSMA), prostate cancer, NeoB, PSMA-617

## Abstract

**Introduction:**

Central to targeted radionuclide imaging and therapy of prostate cancer (PCa) are prostate-specific membrane antigen (PSMA)-targeting radiopharmaceuticals. Gastrin-releasing peptide receptor (GRPR) targeting has been proposed as a potential additional approach for PCa theranostics. The aim of this study was to investigate to what extent and at what stage of the disease GRPR-targeting applications can complement PSMA-targeting theranostics in the management of PCa.

**Methods:**

Binding of the GRPR- and PSMA-targeting radiopharmaceuticals [^177^Lu]Lu-NeoB and [^177^Lu]Lu-PSMA-617, respectively, was evaluated and compared on tissue sections of 20 benign prostatic hyperplasia (BPH), 16 primary PCa and 17 progressive castration-resistant PCa (CRPC) fresh frozen tissue specimens. Hematoxylin-eosin and alpha-methylacyl-CoA racemase stains were performed to identify regions of prostatic adenocarcinoma and potentially high-grade prostatic intraepithelial neoplasia. For a subset of primary PCa samples, RNA *in situ* hybridization (ISH) was used to identify target mRNA expression in defined tumor regions.

**Results:**

The highest median [^177^Lu]Lu-NeoB binding was observed in primary PCa samples, while median and overall [^177^Lu]Lu-PSMA-617 binding was highest in CRPC samples. The highest [^177^Lu]Lu-NeoB binding was observed in 3/17 CRPC samples of which one sample showed no [^177^Lu]Lu-PSMA-617 binding. RNA ISH analyses showed a trend between mRNA expression and radiopharmaceutical binding, and confirmed the distinct GRPR and PSMA expression patterns in primary PCa observed with radiopharmaceutical binding.

**Conclusion:**

Our study emphasizes that GRPR-targeting approaches can contribute to improved PCa management and complement currently applied PSMA-targeting strategies in both early and late stage PCa.

## 1 Introduction

Prostate cancer (PCa) is the second most common male cancer type with 1.4 million new cases and 375.000 deaths worldwide in 2020 (1). In recent years, nuclear medicine has rapidly gained an important status in PCa management. Central to targeted radionuclide imaging and therapy of PCa are prostate-specific membrane antigen (PSMA)-targeting radiopharmaceuticals (2). PSMA is a type II transmembrane glycoprotein that is expressed in normal prostate tissue with significantly increased expression in PCa, especially in advanced stages of the disease (3–6). Following the success of various clinical studies, the radiopharmaceuticals [^68^Ga]Ga-PSMA-11 and [^18^F]F-DCFPyL for positron emission tomography (PET) and [^177^Lu]Lu-PSMA-617 for radionuclide treatment have recently been approved by the FDA and EMA for PCa patients (7–9).

PSMA PET has shown to detect pelvic lymph nodes and metastatic lesions with higher sensitivity and specificity compared to conventional imaging methods such as computed tomography (CT) and bone scintigraphy (10, 11). Regarding treatment with [^177^Lu]Lu-PSMA-617, impressive results were obtained in PSMA-positive metastatic castration-resistant PCa (mCRPC) patients who received [^177^Lu]Lu-PSMA-617 plus standard of care versus standard of care treatment alone; with significantly prolonged progression-free survival and overall survival of 8.7 vs. 3.4 months and 15.3 vs. 11.3 months, respectively (12). Importantly, a proportion (<10%) of prostate carcinomas has low PSMA expression and ~30% of mCRPC patients do not respond to treatment with [^177^Lu]Lu-PSMA-617, when response is defined as any decrease in prostate-specific antigen (PSA) levels (13, 14). Moreover, serious side effects have been frequently reported, such as xerostomia as a consequence of unwanted but specific binding to PSMA in the salivary glands. The impact of xerostomia on patients’ quality of life is the main reason for treatment discontinuation, especially when [^225^Ac]Ac-PSMA-617 is applied (15). All of the above underline the need for new developments with improved efficacy and safety.

The gastrin-releasing peptide receptor (GRPR) is a G-protein coupled receptor that has been investigated as an attractive target for the detection and treatment of several cancer types, including PCa (16). In PCa, overexpression of the GRPR was reported in 63-100% of cases (17). In contrast to PSMA, GRPR overexpression has primarily been associated with low-grade disease (18–22). Initial imaging studies using GRPR-targeting radiopharmaceuticals, such as [^68^Ga]Ga-RM2, [^68^Ga]Ga-RM26, [^68^Ga]Ga-SB3 and [^68^Ga]Ga-NeoBOMB1/NeoB, have shown promising results in the detection of prostate lesions and lymph node metastases of newly diagnosed and recurrent PCa. While these radiopharmaceuticals have mainly been investigated in exploratory studies, the findings suggest that their use, similar to PSMA targeting, could be superior to conventional imaging (23–27). Following the theranostic approach, clinical studies have been initiated to characterize GRPR ligands coupled to a therapeutic radionuclide (e.g. Lu-177 or Pb-212) in various neoplasms (NCT03872778, NCT05283330) (28).

NeoB, formerly called NeoBOMB1, is a GRPR theranostic agent that has been extensively validated in preclinical and initial clinical studies with promising results (27, 29–31). Although studies with GRPR radiopharmaceuticals, including NeoB, have demonstrated high uptake not only in the tumor, but also in the GRPR-expressing pancreas, multiple studies have shown that the pancreas is not expected to be a dose limiting organ for GRPR-mediated treatment (28, 30, 32). The relatively low estimated absorbed dose by the pancreas is most likely due to the rapid washout of the radiopharmaceutical from this organ (29). GRPR-targeting radiopharmaceuticals may therefore offer an advantage over [^177^Lu]Lu-PSMA-617 with respect to safety. The use of GRPR targeting may be of particular importance when radionuclide treatment is considered in earlier stages of PCa, which is currently an active area of research.

Taken together, GRPR-targeting nuclear approaches may complement PSMA targeting in the management of PCa. Therefore, few clinical studies have been initiated making a direct comparison between these two approaches. Since clinical studies are often costly, resource-intensive and time-consuming, investigations are limited to a specific patient population. We believe that preclinical studies can therefore greatly contribute to exploring the potential role of GRPR-targeting applications in the context of the currently applied PSMA targeting for detection and treatment of PCa by studying a broad patient population using the same methodology. To this end, we evaluated and compared *ex vivo* binding of the GRPR- and PSMA-targeting radiopharmaceuticals [^177^Lu]Lu-NeoB and [^177^Lu]Lu-PSMA-617, respectively, to patient tissue sections obtained from benign prostatic hyperplasia (BPH), primary PCa and progressive CRPC lesions.

## 2 Materials and methods

### 2.1 Human prostate specimens

This study adhered to the Code of Conduct of the Federation of Dutch Medical Scientific Societies. Fresh frozen BPH and primary PCa tissue specimens were retrieved from the Erasmus MC Tissue Bank. BPH tissues (adenomyomatous hyperplasia) from 20 patients (mean age ± standard deviation (SD): 69 ± 10 years) obtained after transurethral resection of the prostate (TURP) and primary PCa tissues from 16 patients (65 ± 7 years) obtained from radical prostatectomy were retrieved. Seventeen CRPC fresh frozen samples were selected from the Erasmus MC Urology Department Tissue Biobank and were obtained from TURP of progressive patients treated in hospitals in the Rotterdam region (73 ± 6 years). Patients were included as CRPC when they presented with biochemical or radiological progressive disease after surgical or medical castration. For the BPH and CRPC sample set, 3 different fragments of one TURP per patient were included to study a larger tissue area. Clinicopathological characteristics, such as Gleason score (GS) and PSA, of all PCa patients are summarized in **Tables S1** and **S2**.

### 2.2 Tissue sectioning and staining

Each specimen was cut into 10 µm thick sections and mounted on SuperFrost slides (VWR). Adjacent sections were successively used for autoradiography studies with [^177^Lu]Lu-NeoB (2 sections) and [^177^Lu]Lu-PSMA-617 (2 sections), 1 section was used for hematoxylin-eosin (H&E) staining, in case of primary PCa 1 section was used for Alpha-methylacyl-CoA racemase (AMACR) staining, and 1 section was used for RNA *in situ* hybridization (ISH) analysis (only in a subset). H&E staining was performed according to a standard protocol in order to determine the presence of cancerous areas. Tumor regions were manually drawn by an experienced pathologist (GvL) and graded according to the ISUP 2014 GS (**Table S1**). Immunohistochemistry for AMACR was conducted by the Erasmus MC Pathology Research and Trial Service to identify regions of prostatic adenocarcinoma and potentially premalignant high-grade prostatic intraepithelial neoplasia (PIN), although definitive cytological atypia required for the diagnosis of PIN cannot be established well on frozen sections (33). Staining was performed with an automated, validated and accredited staining system (Ventana Benchmark ULTRA, Ventana Medical Systems) using UltraView Universal DAB Detection Kit. In brief, heat-induced antigen retrieval was performed using the Ventana CC1 solution for 8 min. The tissue samples were then incubated with a monoclonal rabbit anti-AMACR (clone 13H4) for 32 min at a concentration of 1.27 µg/mL. Incubation was followed by hematoxylin II counter stain for 12 min and then a blue coloring reagent for 8 min according to the manufactures instructions (Ventana).

High resolution images of the H&E and AMACR stained sections were acquired using a NanoZoomer digital slide scanner (Hamamatsu Photonics) and analyzed using NDP View 2 software (Hamamatsu Photonics).

### 2.3 Radiopharmaceuticals

The GRPR antagonist NeoB (Advanced Accelerator Applications, a Novartis company) and the PSMA inhibitor PSMA-617 were labeled with lutetium-177 (LuMark, IDB Holland) as described previously (29, 34). Quenchers (ascorbic and gentisic acids) were used to prevent radiolysis (35). High-pressure liquid chromatography and instant thin-layer chromatography on silica gel were used to determine the radiochemical purity (>95%) and radiolabeling yield (>95%) of all labelings. A molar activity of 40 MBq/nmol was used for all *in vitro* autoradiography experiments.

### 2.4 *In vitro* autoradiography

To compare radiopharmaceutical binding, an *in vitro* autoradiography was performed on frozen human prostate sections. Frozen sections of cell line-derived PC-3 (GRPR-positive) and patient-derived PC295 (PSMA-positive) xenograft tumors were used as positive controls (36, 37). In short, tissue sections were incubated for 10 min at room temperature (RT) with washing buffer (167 mM Tris-HCl pH 7.6, 5 mM MgCl_2_) containing 0.25% bovine serum albumin (BSA) to prevent non-specific binding to the glass slides. Tissue sections were subsequently incubated for 1 h at RT with 100 µL of incubation buffer (washing buffer with 1% BSA) containing 1 nM [^177^Lu]Lu-NeoB or [^177^Lu]Lu-PSMA-617 (i.e. total binding). To assess binding specificity, parallel-sections were co-incubated with an excess (1 µM) of unlabeled Tyr^4^-bombesin (Merck Life Science NV) or PSMA-I&T (Huayi Isotopes Co. via ATT Scintomics), respectively. Following incubation, slides were washed and dried before exposure to super-resolution (<50 microns) phosphor screens (Perkin Elmer) for >24 h. Screens were read using the Cyclone (Perkin Elmer) and data was processed in Optiquant software (Perkin Elmer).

[^177^Lu]Lu-NeoB and [^177^Lu]Lu-PSMA-617 binding in the tumor regions, as identified on the adjacent H&E-stained sections, was quantified and expressed as digital light units per surface area (DLU/mm^2^). Standards consisting of 1 µL drops of incubation buffer were quantified to determine the percentage of added activity per mm^2^ (%AA/mm^2^). DLU/mm^2^ was then converted to %AA/mm^2^ by normalizing data to the standards. Specific binding was defined by subtracting the nonspecific binding, as measured on the sections blocked with an excess of unlabeled ligand, from the total binding.

### 2.5 RNA *in situ* hybridization

RNA ISH assay was performed to determine cellular GRPR and PSMA mRNA expression levels which could be correlated to radiopharmaceutical binding. To detect GRPR and PSMA mRNA simultaneously in one sample, ISH was performed using the RNAscope 2.5 HD Duplex Reagent Kit (cat. #322430; Advanced Cell Diagnostics (ACD)) in accordance with the manufacturer’s instructions for the manual chromogenic assay for fresh frozen tissue using optimized sample preparation and pretreatment conditions. Tissues were fixed in pre-chilled 10% neutral buffered formalin (Sigma-Aldrich) for 2 h at 4°C and then rinsed twice with phosphate buffered saline (PBS; ThermoFisher Scientific). After fixation, tissues were dehydrated using a series of ethanol washes and air dried. For tissue pretreatment, sections were exposed to kit-provided hydrogen peroxide for 10 min at RT and then rinsed in PBS. Immediately after, slides were placed in a dry incubator (37°C) for 30 min before being treated with kit-provided protease IV for 30 min at RT. Hybridization of the GRPR probe Hs-GRPR (cat. #460411; ACD) and the newly designed and synthesized PSMA probe Hs-PSMA1-C2 (cat. #311251-C2; ACD) to target respective mRNA sequences was performed by incubation in the HybEZ Oven (cat. #321720; ACD) for 2 h at 40°C. Hybridization was followed by standard signal amplification steps and fast red and green chromogenic detection. Tissues were then counterstained with Gill’s Hematoxylin I (Polysciences Inc.), air dried, mounted and imaged using a NanoZoomer digital slide scanner (Hamamatsu Photonics).

From the whole slide images, regions of interest (ROIs) were selected covering 10% of the tumor area and 5 additional ROIs outside the tumor area to identify non-tumor cell staining. ROIs were manually drawn by a researcher who was blind to the study using QuPath software (38). These ROIs were analyzed using in-house written Python based scripts and graphical interface (Tumor Microenvironment (TME) Analyzer, H.E. Balcioglu, manuscript in preparation), blind to the clinical information (**Figure S1**). Briefly, the bright field images were converted into pseudo-fluorescent images through image inversion followed by manual identification of signal intensity patterns and using these patterns to assign a pixel intensity per signal (i.e. PSMA probe, GRPR probe and nucleus). To remove nonspecific signal detection, signals low in intensity were removed. Additionally, for the GRPR probe, areas larger than 500px in size were removed to overcome the wrong assignment of brown and blue artifacts to this channel. Nuclei were detected by applying StarDist algorithm ‘2D_versatile_fluo’ (39), and cell regions were assigned through Voronoi segmentation up to 50 pixels from the nucleus. Probe positive regions were detected and clusters were defined as large, elongated regions with at least 100 px^2^ size and eccentricity of at least 0.7. Cells were then assigned to 5 bins: bin 0, no probe positivity; bin 1, probe positivity but no clusters; bin 2, 1 probe cluster; bin 3, 2 probe clusters; bin 4, at least 3 probe clusters. H-scores were calculated as follows: $\text{H-score}=\sum_{\begin{array}{c} \text{bin}\\ 0\to 4 \end{array}}\text{(bin number x percentage of cells per bin)}$ .

### 2.6 Statistics

All statistical analyses were carried out using GraphPad Prism software, version 9 (GraphPad Software Inc.). The distribution of radiopharmaceutical binding within disease stages was depicted in violin plots. When multiple samples from the same patient were available, only one randomly selected sample was used for statistics. As the Shapiro-Wilk test indicated that data was not normally distributed, the nonparametric Kruskal-Wallis test followed by Dunn’s *post-hoc* test for multiple comparisons was performed to compare mean GRPR or PSMA radiopharmaceutical binding between disease stages, and between ISUP grades for primary PCa. A *p* value of <0.05 was considered statistically significant. Pearson’s correlation coefficient was determined to measure the association between radiopharmaceutical binding and mRNA expression.

## 3 Results

### 3.1 Binding pattern

**Figure 1:**
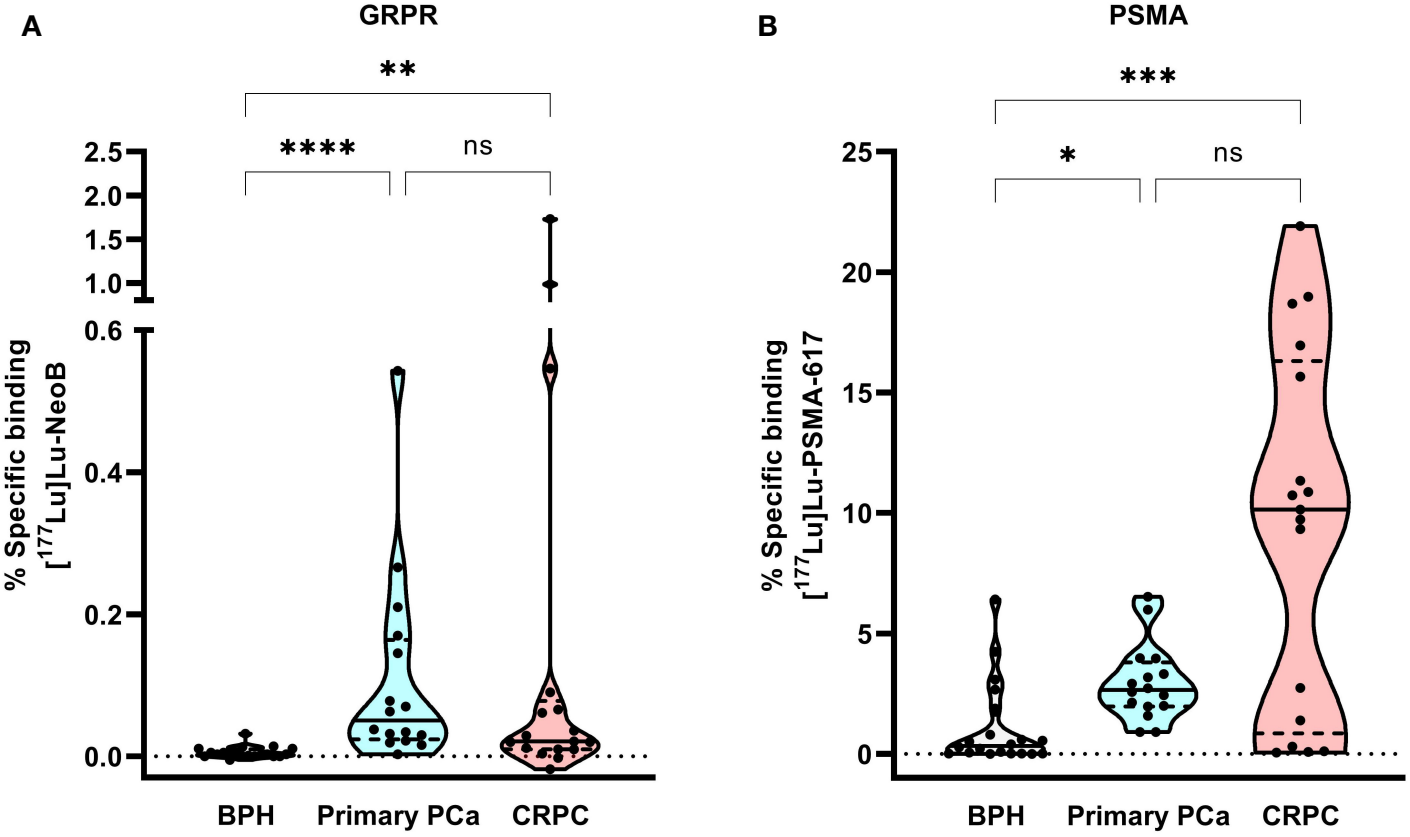
Violin plot of the percentage of [^177^Lu]Lu-NeoB **(A)** and [^177^Lu]Lu-PSMA-617 **(B)** specific binding to the same set of benign prostatic hyperplasia (BPH; n=20), primary prostate cancer (PCa) (n=16) and castration-resistant PCa (CRPC; n=17) patient samples. The black line represents the median, the dashed lines the two quartiles and the dotted line the 0 axis. Percentage specific binding represents the percentage of added activity per mm^2^. * = *p* < 0.05, ** = *p* < 0.01 and **** = *p* < 0.0001, GRPR, gastrin-releasing peptide receptor; ns, not significant; PSMA, prostate-specific membrane antigen.

**Figure 1** shows the percentage specific binding of [^177^Lu]Lu-NeoB and [^177^Lu]Lu-PSMA-617 for the individual BPH, primary PCa and CRPC samples. The highest median [^177^Lu]Lu-NeoB binding (0.051% AA/mm^2^, 95% confidence interval (CI) = 0.034 – 0.183%) was observed in primary PCa samples, while median [^177^Lu]Lu-PSMA-617 binding (10.74% AA/mm^2^, 95% CI = 5.52 – 13.19%) was highest in CRPC samples. Binding of both radiopharmaceuticals was specific for PCa, as the mean percentage specific binding in both primary PCa and CRPC samples was significantly higher than in BPH (*p* < 0.05). The results of the correlational analysis determined no significant difference in radiopharmaceutical binding by ISUP grade in primary PCa for either [^177^Lu]Lu-NeoB or [^177^Lu]Lu-PSMA-617.

The percentage specific binding of [^177^Lu]Lu-PSMA-617 in 4/20 BPH samples was within or above the upper limit of the 95% CI of binding in primary PCa, indicating relatively high PSMA expression levels in these samples. In contrast, for [^177^Lu]Lu-NeoB binding this was not true for any of the BPH samples. The highest variation of binding of both radiopharmaceuticals was observed within the CRPC sample set, illustrating that there is a high degree of heterogeneity in GRPR and PSMA expression between patients with advanced disease. Although [^177^Lu]Lu-PSMA-617 binding was generally high in CRPC samples, 6/17 samples showed no or very low binding (i.e. binding below the lower limit of the 95% CI). Interestingly, one of these samples showed relatively high [^177^Lu]Lu-NeoB binding. Moreover, 3/17 CRPC samples showed a high level of [^177^Lu]Lu-NeoB binding that falls outside the 95% probability limit of binding in primary PCa.

### 3.2 Intrapatient heterogeneity

**Figure 2:**
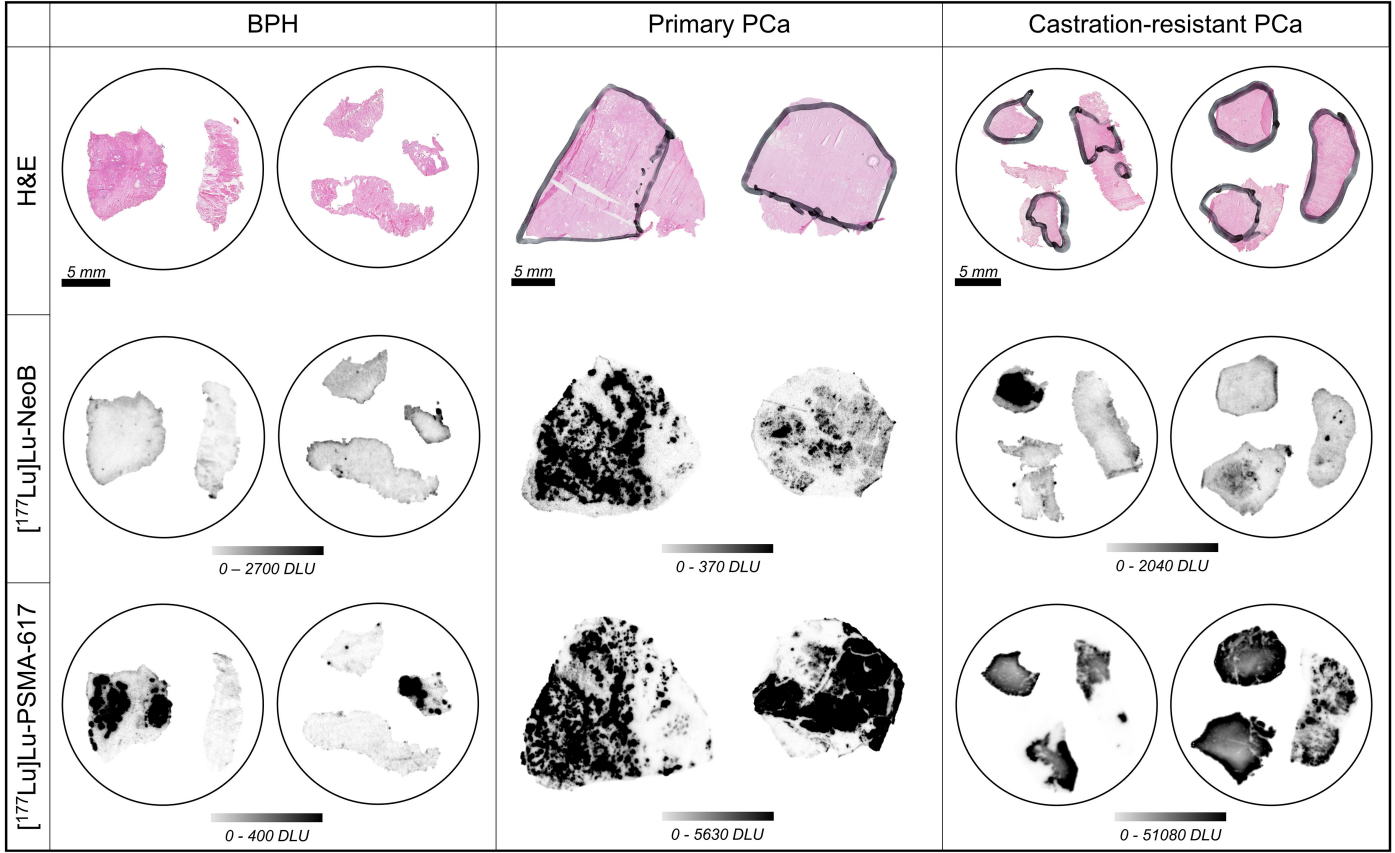
Hematoxylin-eosin (H&E) stains (top row), binding of [^177^Lu]Lu-NeoB (middle row) and [^177^Lu]Lu-PSMA-617 (bottom row) to representative samples of benign prostatic hyperplasia (BPH), primary prostate cancer (PCa) and castration-resistant PCa. Encircled tissue sections belong to the same patient. The black marking in the H&E stained sections indicates tumor area(s) as identified by a pathologist. The scale selected for the autoradiography sections shows optimized contrast. DLU, digital light unit.

Next to interpatient heterogeneity, we observed large intrapatient heterogeneity for both [^177^Lu]Lu-PSMA-617 and [^177^Lu]Lu-NeoB binding across the various disease states. This intrapatient heterogeneity was reflected in differences in signal intensity between various locations within the prostate and within the tumor region of one section (**Figure 2**). In order to study the heterogeneity between various locations within the prostate, fragments from different sites in the prostate or prostate tumor of the same patient were analyzed for the BPH and CRPC stages, respectively. It was observed that not all samples from the same patient showed binding or showed the same degree of binding.

### 3.3 Tumor specificity

**Figure 3:**
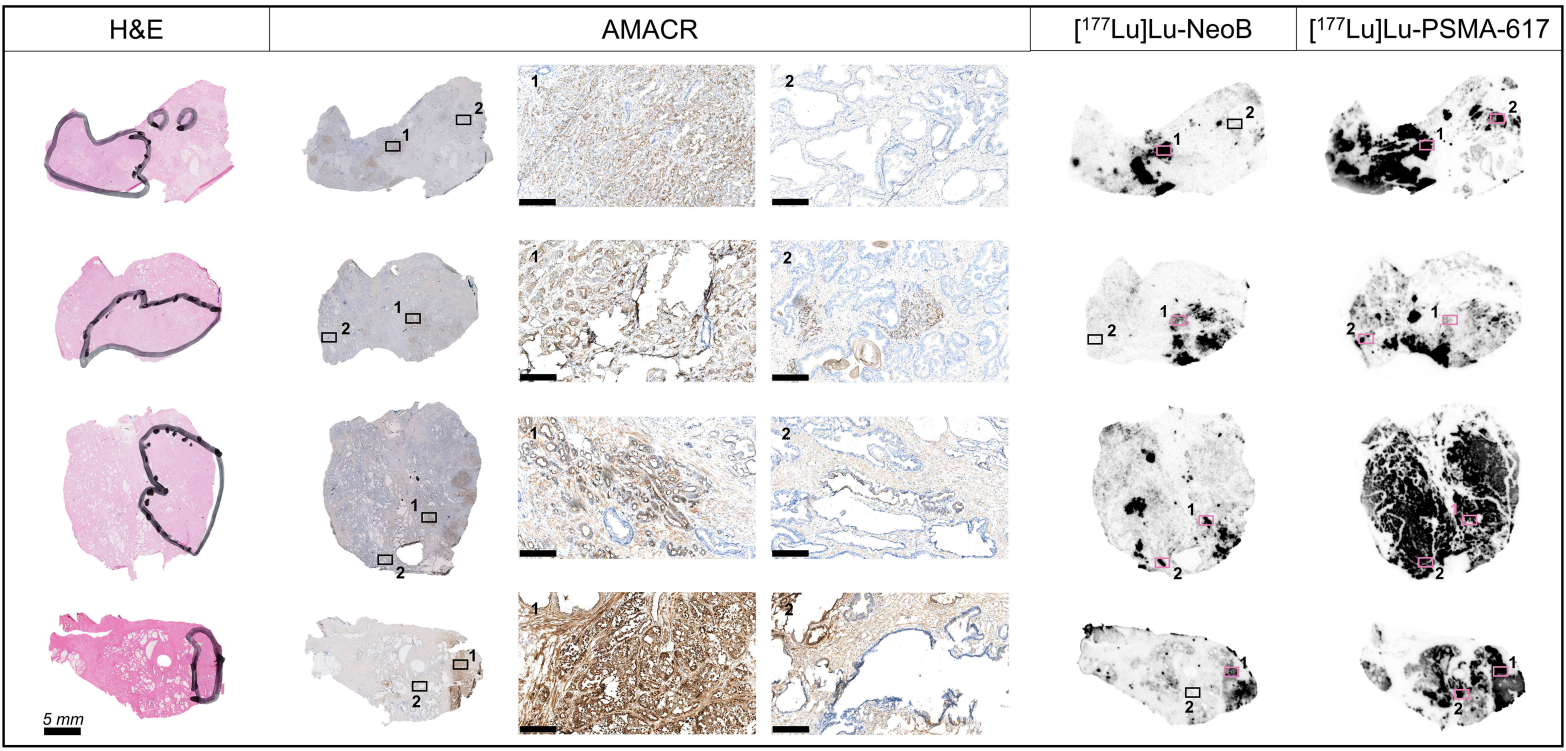
Tumor specificity of [^177^Lu]Lu-NeoB and [^177^Lu]Lu-PSMA-617 in primary prostate cancer sections of four representative patients. From left to right in the figure: hematoxylin-eosin (H&E) staining, immunohistochemical staining of alpha-methylacyl-CoA racemase (AMACR) expression, [^177^Lu]Lu-NeoB and [^177^Lu]Lu-PSMA-617 binding. The black marking in the H&E stained sections indicates the tumor area(s) as identified by a pathologist. For the AMACR stained section, a 10x magnification of two specified areas (black/pink box numbered 1 or 2) is shown (scale bar = 250 µm). The presented autoradiography sections are displayed at an optimal scale for each tissue to show optimized contrast.

Analysis of tumor specificity was conducted in the relatively larger sections of the primary PCa samples using H&E and AMACR stainings to detect prostatic adenocarcinoma cells. This analysis showed that binding of [^177^Lu]Lu-PSMA-617 and [^177^Lu]Lu-NeoB occurred in a focal pattern that corresponded with AMACR staining intensity (**Figure 3**). Small areas of AMACR positively stained cells were observed within and outside the tumor regions identified by H&E supported pathology. In the majority of cases, these AMACR positive areas outside of the tumor region also showed relatively high [^177^Lu]Lu-PSMA-617 and [^177^Lu]Lu-NeoB binding and may represent premalignant high-grade PIN. Interestingly, in 5/16 cases (3/5 ISUP grade 2, 2/5 ISUP grade 3) high [^177^Lu]Lu-PSMA-617 binding and in contrast no or very little [^177^Lu]Lu-NeoB binding was observed to cells outside the tumor region and AMACR positively stained areas (i.e. normal tissue). Here, histological evaluation revealed the presence of normal epithelial cells lining glandular ducts. The high [^177^Lu]Lu-PSMA-617 binding was target specific as no binding was observed in the blocked section (**Figure S2**).

### 3.4 mRNA expression

**Figure 4:**
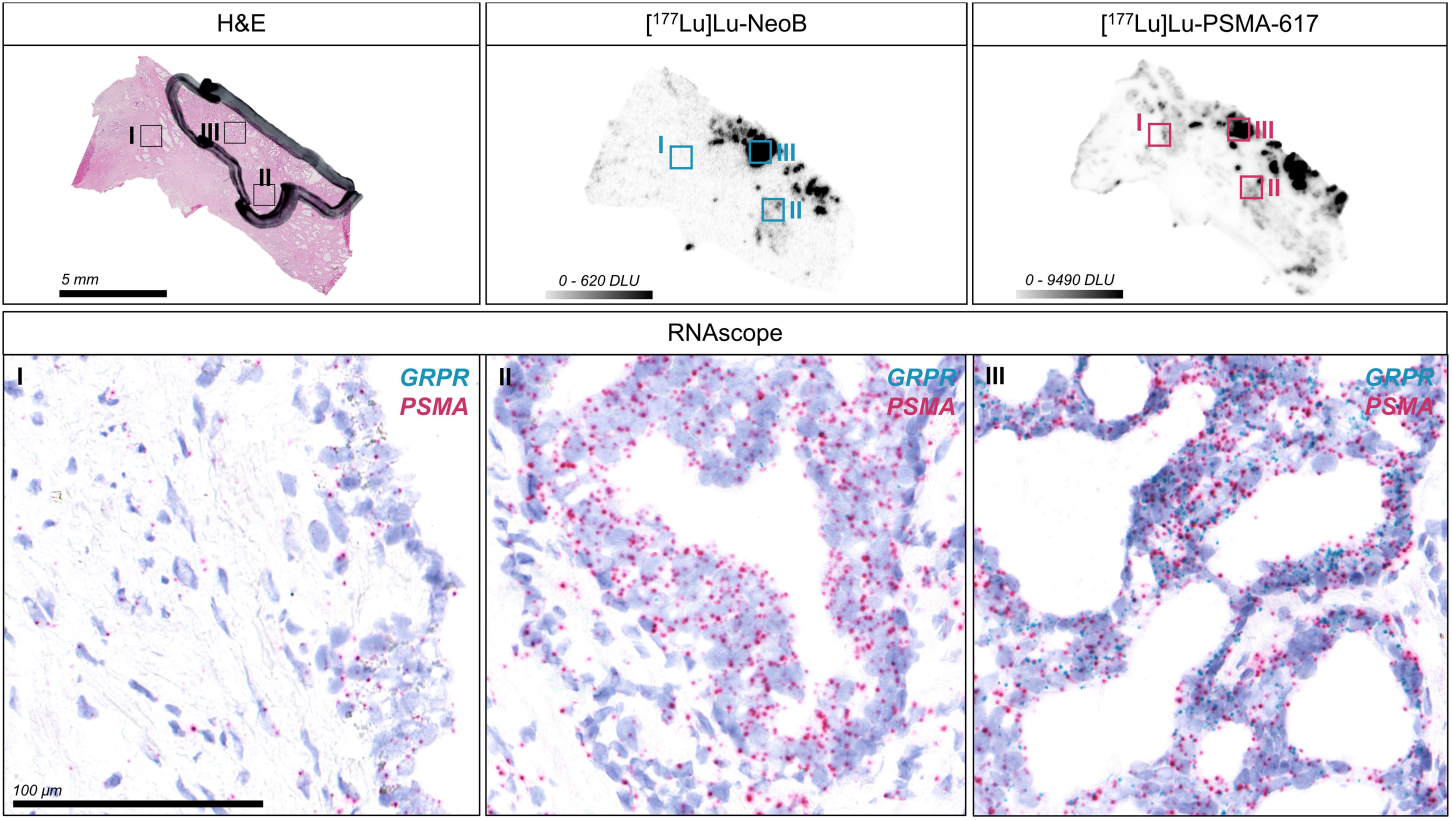
Detection of gastrin-releasing peptide receptor (GRPR) and prostate-specific membrane antigen (PSMA) mRNA expression in one representative primary prostate cancer section using RNA *in situ* hybridization (ISH). The hematoxylin-eosin (H&E) stained section with black marking indicates the tumor area as identified by a pathologist. The corresponding autoradiography images for [^177^Lu]Lu-NeoB and [^177^Lu]Lu-PSMA-617 binding are displayed and the selected color scale shows the optimized contrast for the sections. Forty times magnifications of the RNA ISH section showing a region with background [^177^Lu]Lu-NeoB and [^177^Lu]Lu-PSMA-617 binding in non-tumor tissue (I) and with relatively low (II) and high (III) binding within the tumor are shown. Each dot represents a single GRPR (blue) or PSMA (red) mRNA molecule. Nuclei were counterstained with hematoxylin (purple). DLU, digital light unit.

An RNA ISH assay was conducted to evaluate the relation between mRNA expression and radiopharmaceutical binding (**Figure 4**, **Figure S3**). Although significance was not observed, probably due to the low number of samples, PSMA mRNA expression levels (expressed as H-score) showed a trend that correlated positively with radiopharmaceutical binding (*n* = 5; r = 0.64; p = 0.25). No strong trend was found for GRPR (*n* = 5; r = 0.34; p = 0.57). In line with the results of the autoradiography studies, the H-score for PSMA was significantly higher than for GRPR (mean ± SD; 72.4 ± 18.1 for PSMA vs. 10.0 ± 5.4 for GRPR; *p* < 0.01), indicating higher PSMA expression (**Table S3**). PSMA mRNA levels in non-tumor areas were also significantly higher than GRPR mRNA levels (*p* < 0.05) (**Table S4**). Moreover, analysis showed that of all target-positive cells there was a considerable proportion of single positive cells (range; 76.7-92.8% and 12.4-43.9% for PSMA and GRPR positive cells, respectively), reflecting the distinct expression patterns of PSMA and GRPR.

## 4 Discussion

PSMA-targeting radiopharmaceuticals have emerged as powerful agents for PCa management. However, not all PCa patients have PSMA overexpression and a considerable proportion of PSMA-positive patients does not respond to treatment with [^177^Lu]Lu-PSMA-617. Moreover, PSMA targeting comes with serious side effects as the result of unwanted but specific binding to PSMA on the salivary glands. This calls for improved PCa theranostics by, for example, using other targets. In this study, we examined whether GRPR-targeting radiopharmaceuticals can complement PSMA-targeting theranostic approaches and where to position them in the progression of PCa. We compared [^177^Lu]Lu-NeoB and [^177^Lu]Lu-PSMA-617 binding in patient samples of BPH, primary PCa and CRPC using the same methodology across all stages. Whilst some clinical research has been carried out on such a comparison, in this preclinical research we were able to cover a broad range of prostate conditions thereby our study contributes to an increased understanding of the link between radiopharmaceutical binding and disease stage.

The data showed the highest median binding of [^177^Lu]Lu-NeoB in primary PCa samples, while the highest median binding of [^177^Lu]Lu-PSMA-617 was observed in CRPC samples. This finding confirms the work of others, in which high GRPR and PSMA expression were linked to the respective disease stages (5, 18). Although, in contrast to our previous report on breast cancer (40), we did not observe a statistically significant relationship between radiopharmaceutical binding and target mRNA expression, we expect this to be due to sample size rather than different tissue type. Even though we found the highest median binding of [^177^Lu]Lu-NeoB in primary PCa, the 3 samples with the highest [^177^Lu]Lu-NeoB binding belonged to the CRPC sample set. This may suggest that GRPR-targeting approaches may be relevant in a small proportion of patients with late stage disease.

We observed that 1/6 PSMA-negative CRPC samples showed high [^177^Lu]Lu-NeoB binding, indicating a potential complementary role of GRPR targeting to PSMA theranostics. Although these are low numbers, Baratto et al. (41) also underlined the complementary value of GRPR targeting as they identified 7 additional lesions with [^68^Ga]Ga-RM2 (a potent GRPR-targeting agent) PET that were not visible on [^68^Ga]Ga-PSMA11/[^18^F]F-DCFPyL PET in 4/50 biochemically recurrent PCa patients. Kurth et al. (28) investigated 35 patients with metastatic CRPC who had insufficient PSMA expression or showed lower tumor accumulation after previous cycles of [^177^Lu]Lu-PSMA-617 treatment. They identified 6 patients with high uptake on [^68^Ga]Ga-RM2 PET/CT and who thus qualified for [^177^Lu]Lu-RM2 therapy. In their study, the absorbed doses in the tumor lesions delivered by [^177^Lu]Lu-RM2 were found to be therapeutically relevant. Taken together, our findings support that a subset of metastatic CRPC patients might benefit from GRPR-mediated radionuclide therapy.

Although [^177^Lu]Lu-PSMA-617 radionuclide therapy is currently only available for patients with metastatic CRPC, further studies are ongoing to explore its use in earlier stages of PCa. In our study, we observed binding of both [^177^Lu]Lu-NeoB and [^177^Lu]Lu-PSMA-617 in all but one primary PCa samples. This finding is consistent with that of Mapelli et al. (42) who reported detection of primary PCa in 18/19 patients with both GRPR- and PSMA-targeting radiopharmaceuticals separately. A limitation for the use of PSMA radiopharmaceuticals in primary PCa is the relatively high binding of [^177^Lu]Lu-PSMA-617 to BPH samples and normal tissue surrounding primary PCa with ISUP grade 2 and 3, as observed in our study. Our results indicate that PSMA-targeting applications, in contrast to GRPR, may not always distinguish cancerous tissue from benign or normal tissue, reducing the tumor-specific value. This is one of the pitfalls of PSMA PET that has also been described before (43).

Analyzing radiopharmaceutical binding in primary PCa within the ISUP grades for PCa classification revealed no significant differences between grades. However, prior preclinical studies evaluating the expression of GRPR and PSMA in PCa samples have reported on a higher GRPR expression for low-grade PCa specimens. Faviana et al. (44) found that the number of cells expressing GRPR as determined by immunohistochemistry was significantly higher in low-grade tumors and Schollhammer et al. (45) demonstrated higher [^111^In]In-RM2 binding in primary PCa samples with Gleason score 6 (i.e. ISUP grade 1) using autoradiography studies. The absence of a found correlation in our study may be due to the unequal distribution of samples across the 5 ISUP grade groups in combination with the small sample size reducing the statistical power. Unlike the aforementioned studies, our primary objective was to compare expression levels across the different disease stages and thus ISUP grade was not taken into account when samples were selected. Similarly as our study contrasts with other preclinical reports, clinical studies reported contradictory results as well; Gao et al. (46) noted higher uptake in low-ISUP PCa, while Schollhammer et al. (47) saw no differences in uptake between ISUP grades for GRPR-mediated PET/CT. More research is needed to get a clear answer on the association between ISUP grade and GRPR expression levels in primary PCa.

For primary PCa, the complementary value of GRPR targeting may also be found in the fact that we observed binding of [^177^Lu]Lu-NeoB and [^177^Lu]Lu-PSMA-617 to overlapping, but also to different tumor areas within the tumor region of one sample. This finding of distinct GRPR and PSMA expression patterns was further supported by our RNA ISH results indicating differential mRNA expression of these targets per cell. There was a considerable proportion of single positive cells that showed mRNA signal only for GRPR or PSMA, although GRPR positivity might be underestimated due to color overlap with the cell nucleus. This complementary role for GRPR-targeting radiopharmaceuticals based on the different expression patterns has also been suggested in previous studies (48, 49). The observed intrapatient heterogeneity of GRPR and PSMA suggests that future theranostics for primary prostate tumors may benefit from an approach in which GRPR- and PSMA-targeting radiopharmaceuticals are combined. One such approach currently being explored by various research groups is the use of GRPR/PSMA-targeting heterodimers (50–52). Reported results so far are preliminary and the value of such heterodimers remains to be investigated. Of note, if GRPR/PSMA heterodimers will be applied for radionuclide therapy, the off-target organ toxicity should critically be evaluated as GRPR and PSMA are physiologically expressed on different background organs, which may increase toxicity.

The generalizability of these results is subject to limitations. While autoradiography studies provide a direct measurement of radiopharmaceutical binding and allow for the high-resolution visualization of binding, this does not reflect *in vivo* pharmacokinetics. The large difference in specific binding of [^177^Lu]Lu-NeoB and [^177^Lu]Lu-PSMA-617 observed in our study could partly be attributed to this, as the difference in SUVmax in PET studies is generally much smaller. Furthermore, because of the heterogeneous CRPC sample set, correlations with clinicopathological parameters were not addressed in this study. With regard to the RNA ISH analysis, only a single primary PCa section was analyzed per patient for a limited number of patients, limiting the statistical significance of our findings. Therefore, given the general knowledge of tumor heterogeneity, we should interpret our results with caution. Despite these limitations, our study contributes to the knowledge of GRPR and PSMA expression profiles across PCa disease stages and their use as potential targets for theranostic applications.

## 5 Conclusion

Our study demonstrates that GRPR-targeting radiopharmaceuticals may have complementary value for a theranostic approach in both early and late stages of PCa. Furthermore, we showed that relatively high PSMA binding, in contrast to GRPR binding, may be non-tumor specific at early stage PCa. Our study contributes to a better understanding of how to position GRPR targeting in the context of PSMA-directed PCa theranostics to ultimately advance clinical care for PCa patients.

## Data availability statement

The original contributions presented in the study are included in the article/**Supplementary Material**. Further inquiries can be directed to the corresponding author.

## Ethics statement

Ethical review and approval was not required for the study on tissue specimens of human participants in accordance with the local legislation and institutional requirements. The patients/participants provided their written informed consent to participate in this study.

## Author contributions

Conceptualization: SD. Design: WW and SD. Data acquisition: MV, ER and LB. Data analysis: MV, ER, GL, LB and HB. Data interpretation: MV, ER, WW and SD. Writing the first draft of the manuscript: MV. Critically reviewing the manuscript: ER, GL, HB, WW and SD. All authors read and approved the submitted version.
