# Supplementary material for: GRPR versus PSMA: expression profiles during prostate cancer progression demonstrate the added value of GRPR-targeting theranostic approaches
Source: Front Oncol. 2023 Aug 31;13:1199432. doi: 10.3389/fonc.2023.1199432 (PMC10502172; doi:10.3389/fonc.2023.1199432)
Supplement: Supplementary file 1 [file DataSheet_1.docx]

Supplementary Material

GRPR versus PSMA: Expression Profiles During Prostate Cancer Progression Demonstrate the Added Value of GRPR-targeting Theranostic Approaches

Marjolein Verhoeven, Eline A.M. Ruigrok, Geert J.L.H. van Leenders, Lilian van den Brink, Hayri E. Balcioglu, Wytske M. van Weerden and Simone U. Dalm^*^

*** Correspondence:** Simone U. Dalm: s.dalm@erasmusmc.nl

# Supplementary Tables and Figures

## Supplementary Tables

**Supplementary Table 1.** Clinicopathological characteristics of patients with primary PC included in this study.

| **Patient** | **Age (years)** | **PSA at diagnosis (ng/mL)** | **TNM stage at diagnosis** | **Gleason score section** | **ISUP grade** |
| --- | --- | --- | --- | --- | --- |
| 1 | 65 | 6.6 | pT3 | 7 (4+3) | 3 |
| 2 | 71 | 84 | pT3 | 7 (4+3) | 3 |
| 3 | 62 | 24.8 | pT2 | 7 (3+4) | 2 |
| 4 | 70 | 6.2 | pT3 | 9 (5+4) | 5 |
| 5 | 64 | 17 | pT3 | 6 (3+3) | 1 |
| 6 | 53 | 12.1 | pT2 | 7 (4+3) | 3 |
| 7 | 54 | 29 | pT3 | 7 (3+4) | 2 |
| 8 | 73 | 8.4 | pT3 | 7 (3+4) | 2 |
| 9 | 72 | NA | pT3 | 7 (3+4) | 2 |
| 10 | 68 | 11.6 | pT3 | 6 (3+3) | 1 |
| 11 | 72 | 6.9 | pT3 | 8 (4+4) | 4 |
| 12 | 54 | 6.5 | pT3 | 7 (4+3) | 3 |
| 13 | 62 | 6.3 | pT2 | 7 (4+3) | 3 |
| 14 | 65 | 10.3 | pT3 | 6 (3+3) | 1 |
| 15 | 70 | 8.6 | pT2 | 7 (3+4) | 2 |
| 16 | 66 | 4 | pT2 | 7 (3+4) | 2 |

ISUP = International Society of Urological Pathology; NA = not available; PSA = prostate-specific antigen; TNM = tumor (T), nodes (N), and metastases (M)

**Supplementary Table 2.** Clinicopathological characteristics of patients with progressive castration-resistant PC included in this study. PSA values were determined prior to surgery. Prior treatments are listed in chronological order.

| **Patient** | **Age (years)** | **PSA (ng/mL)** | **TNM stage at diagnosis** | **Gleason score at diagnosis** | **ISUP grade** | **Prior treatments** |
| --- | --- | --- | --- | --- | --- | --- |
| 1 | 76 | 47 | NA | 10 (5+5) | 5 | ADT, RT, Chemotherapy, RT, Antiandrogens |
| 2 | 66 | 468.2 | NA | NA |  | ADT + Antiandrogen, Chemotherapy, TKI, Antiandrogen |
| 3 | 83 | 61 | NA | NA |  | RT, ADT, Chemotherapy, Antiandrogen |
| 4 | 75 | 35 | cT3N+Mx | 5 (2+3) | 1 | ADT, RT, Chemotherapy |
| 5 | 68 | NA | NA | NA |  | ADT, Chemotherapy |
| 6 | 79 | 90 | NA | 7 (3+4) | 2 | PLND, ADT + Antiandrogen, Antiandrogen |
| 7 | 81 | 7.3 | cT4N1M1a | 8 (4+4) | 4 | ADT + Antiandrogen |
| 8 | 78 | 85 | cT3N1Mx | 7 (3+4) | 2 | Antiandrogen, ADT, Chemotherapy, Antiandrogen |
| 9 | 64 | NA | cT3N1M1 | 7 (4+3) | 3 | ADT, Chemotherapy |
| 10 | 66 | NA | cT3aN1M1a | 9 (4+5) | 5 | ADT |
| 11 | 72 | 32 | cT1cN0M1b | 8 (4+4) | 4 | ADT |
| 12 | 73 | 46 | cTxNxM1 | NA |  | ADT |
| 13 | NA | NA | cT2-3NxM1 | 7 (4+3) | 3 | Antiandrogen, ADT + Antiandrogen |
| 14 | 70 | 39.5 | cT2aN1M1 | 9 (5+4) | 5 | ADT + Antiandrogen, Chemotherapy, ADT, Antiandrogen, Chemotherapy |
| 15 | 74 | 13.9 | cT4N1M0 | 9 (4+5) | 5 | ADT + Antiandrogen, RT |
| 16 | 73 | 130 | cTxNxM1 | NA |  | RT, ADT |
| 17 | 67 | 4.5 | cT3aN1M1a | 9 (4+5) | 5 | ADT |

ADT = androgen depletion therapy through GnRH agonist/antagonist or orchiectomy; ISUP = International Society of Urological Pathology; NA = not available; PLND = pelvic lymph node dissection; PSA = prostate-specific antigen; RT = radiotherapy; TKI = tyrosine kinase inhibitor; TNM = tumor (T), nodes (N), and metastases (M)

**Supplementary Table 3.** RNAscope analysis results of 10% of the tumor area of a subset of primary PCa patient samples (*n* = 5). The H-score is calculated by multiplying the percentage of cells per bin by the bin number and adding this value from each bin.

|  | Patient 2 | | Patient 4 | | Patient 5 | | Patient 12 | | Patient 13 | |
| --- | --- | --- | --- | --- | --- | --- | --- | --- | --- | --- |
|  | GRPR | PSMA | GRPR | PSMA | GRPR | PSMA | GRPR | PSMA | GRPR | PSMA |
| % Cells in bin 0 | 94.3 | 30.6 | 94.5 | 50.5 | 90.7 | 61.3 | 90.3 | 48.6 | 80.9 | 28.2 |
| % Cells in bin 1 | 5.7 | 52.3 | 5.4 | 33.4 | 9.2 | 32.3 | 9.4 | 37.3 | 18.9 | 55.8 |
| % Cells in bin 2 | 0.0 | 14.8 | 0.1 | 13.2 | 0.1 | 5.8 | 0.3 | 12.3 | 0.2 | 14.2 |
| % Cells in bin 3 | 0.0 | 2.0 | 0.0 | 2.4 | 0.0 | 0.5 | 0.0 | 1.5 | 0.0 | 1.4 |
| % Cells in bin 4 | 0.0 | 0.3 | 0.0 | 0.6 | 0.0 | 0.1 | 0.0 | 0.3 | 0.0 | 0.3 |
| **H-score** | **6** | **89** | **6** | **69** | **9** | **46** | **10** | **68** | **19** | **90** |
| *Co-localization of targets:* | | | | | | | | | | |
| % of positive cells | 5.7 | 69.4 | 5.5 | 49.5 | 9.3 | 38.7 | 9.7 | 51.4 | 19.1 | 71.8 |
| % single positive cells | 0.7 | 64.4 | 1.7 | 45.6 | 4.1 | 33.4 | 2.9 | 44.7 | 2.4 | 55.1 |
| % dual positive cells | 5.0 | | 3.9 | | 5.2 | | 6.8 | | 16.7 | |
| % dual negative cells | 29.9 | | 48.8 | | 57.3 | | 45.6 | | 25.8 | |

**Supplementary Table 4.** RNAscope analysis results of 5 non-tumor regions of interests of a subset of primary PCa patient samples (*n* = 5). The H-score is calculated by multiplying the percentage of cells per bin by the bin number and adding this value from each bin.

|  | Patient 2 | | Patient 4 | | Patient 5 | | Patient 12 | | Patient 13 | |
| --- | --- | --- | --- | --- | --- | --- | --- | --- | --- | --- |
|  | GRPR | PSMA | GRPR | PSMA | GRPR | PSMA | GRPR | PSMA | GRPR | PSMA |
| % Cells in bin 0 | 86.9 | 43.6 | 97.1 | 71.5 | 93.9 | 84.0 | 96.7 | 41.0 | 92.5 | 50.2 |
| % Cells in bin 1 | 13.1 | 44.1 | 2.8 | 24.4 | 6.1 | 15.4 | 3.3 | 42.8 | 7.4 | 40.9 |
| % Cells in bin 2 | 0.0 | 11.2 | 0.0 | 3.4 | 0.1 | 0.6 | 0.0 | 14.7 | 0.0 | 8.1 |
| % Cells in bin 3 | 0.0 | 1.0 | 0.0 | 0.5 | 0.0 | 0.0 | 0.0 | 1.4 | 0.0 | 0.7 |
| % Cells in bin 4 | 0.0 | 0.2 | 0.0 | 0.1 | 0.0 | 0.0 | 0.0 | 0.1 | 0.0 | 0.1 |
| **H-score** | **13** | **70** | **3** | **33** | **6** | **17** | **3** | **77** | **8** | **60** |
| *Co-localization of targets:* | | | | | | | | | | |
| % of positive cells | 13.1 | 56.4 | 2.9 | 28.5 | 6.1 | 16.0 | 3.3 | 59.0 | 7.5 | 49.8 |
| % single positive cells | 3.3 | 46.6 | 1.0 | 26.6 | 3.7 | 13.5 | 0.6 | 56.3 | 2.1 | 44.5 |
| % dual positive cells | 9.8 | | 1.8 | | 2.4 | | 2.7 | | 5.4 | |
| % dual negative cells | 40.3 | | 70.5 | | 80.3 | | 40.3 | | 48.1 | |

##
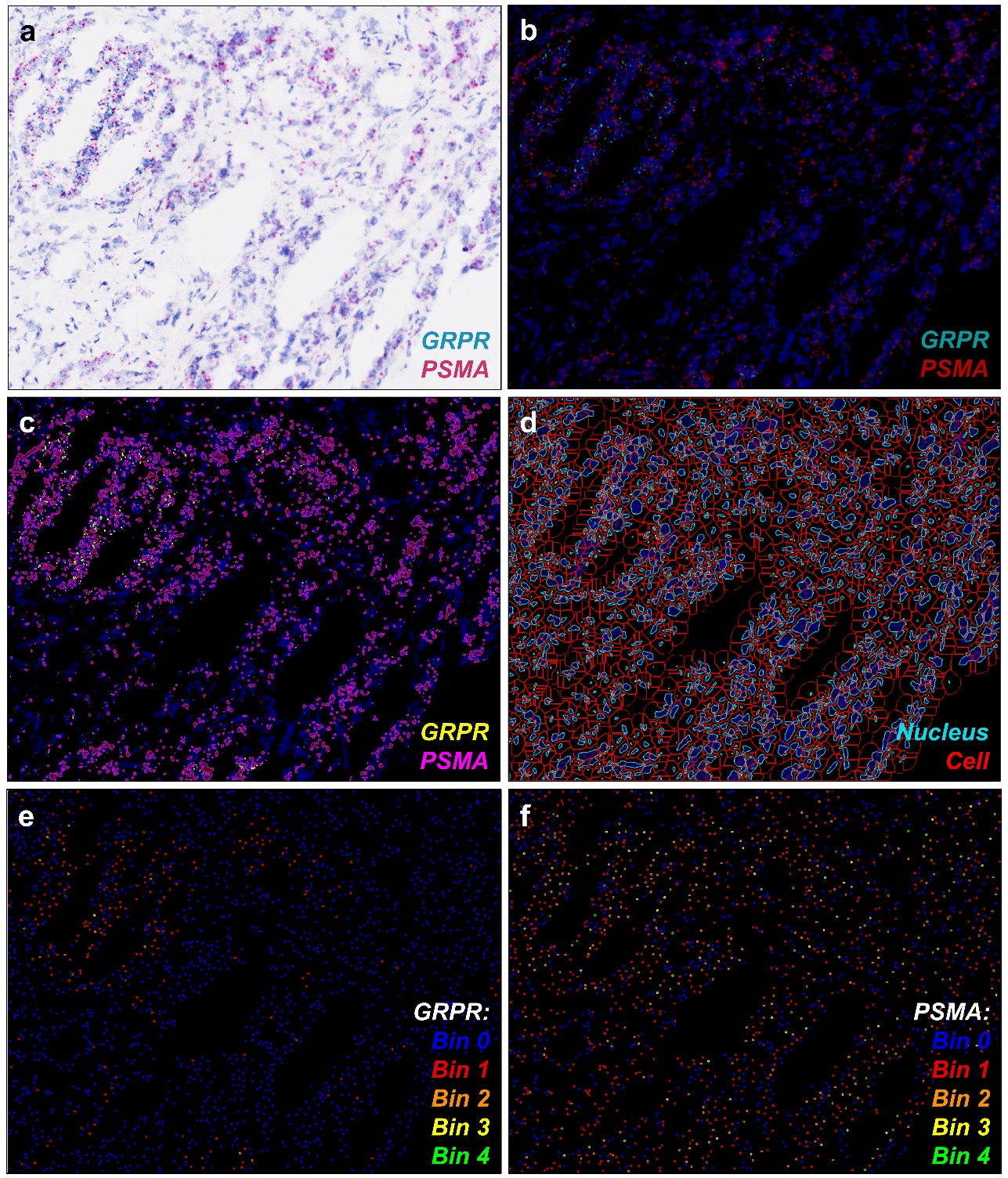
Supplementary Figures

**Supplementary Figure 1.** Signal segmentation of digitalized RNAscope sections for one example region of interest. The digitalized original image (a) was converted into a pseudo-fluorescent image (b) which was used to detect GRPR (yellow) and PSMA (magenta) probe regions (c), and to perform cell segmentation (d) with the nuclear boundaries shown in blue and the cell boundaries in red. Cell binning was performed for both GRPR (e) and PSMA (f), with each cell assigned to a bin: bin 0, no probe positivity (blue); bin 1, probe positivity but no clusters (red); bin 2, 1 probe cluster (orange); bin 3, 2 probe clusters (yellow); bin 4, at least 3 probe clusters (green). A cluster was defined as a large, elongated region with at least 100 px^2^ size and an eccentricity of ≥ 0.7.


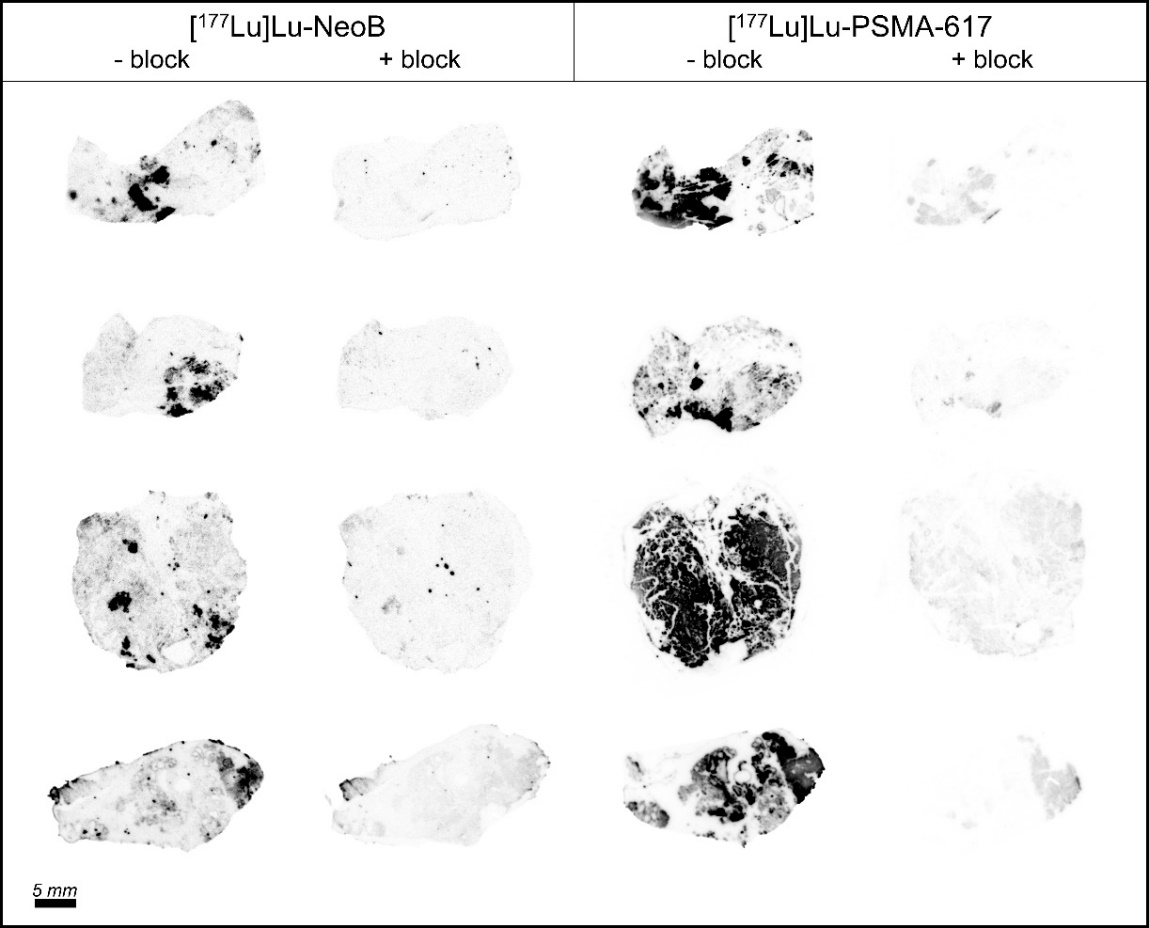


**Supplementary Figure 2.** Target specificity of [^177^Lu]Lu-NeoB and [^177^Lu]Lu-PSMA-617 on primary prostate cancer sections of four representative patients. Binding of [^177^Lu]Lu-NeoB (left) and [^177^Lu]Lu-PSMA-617 (right) in the absence (- block) or presence (+ block) of an excess of unlabeled Tyr4-bombesin or PSMA-I&T, respectively. The presented autoradiography sections each have a different color scale to show optimized contrast.


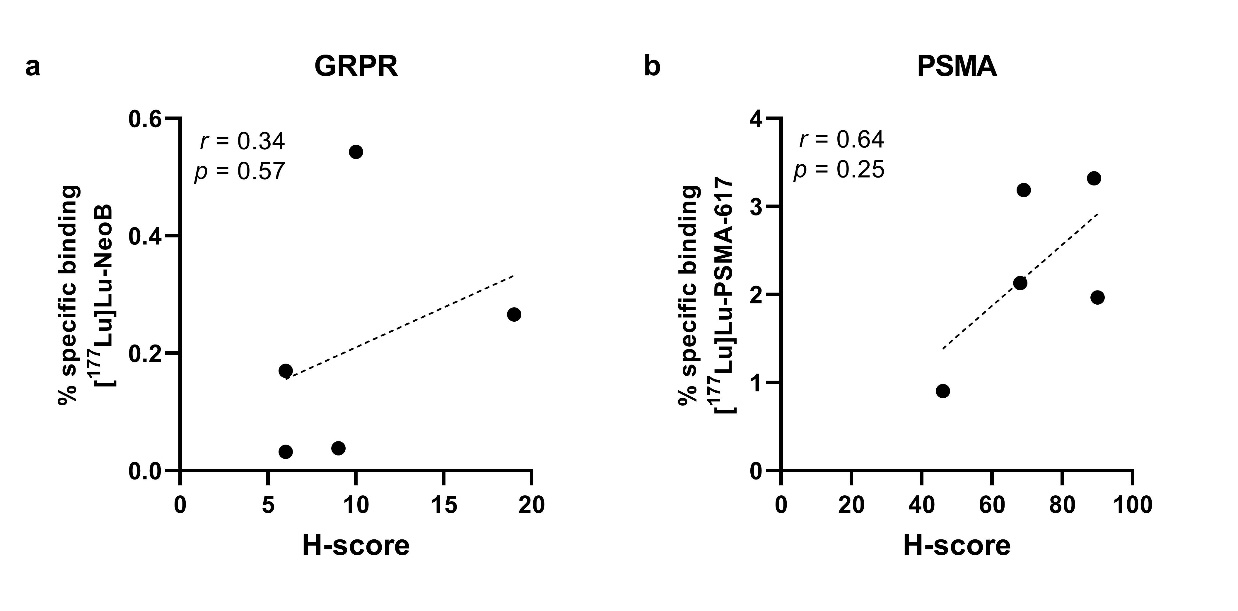


**Supplementary Figure 3.** Correlation of the percentage of [^177^Lu]Lu-NeoB (A) and [^177^Lu]Lu-PSMA-617 (B) specific binding with GRPR and PSMA mRNA expression levels (expressed as H-score), respectively, for the same set of fresh frozen primary PCa samples (*n* = 5). The black dotted line represents the simple linear regression fit where Pearson’s *r* represents the strength of the linear relationship and *p* represents the corresponding p-value.
